# The association between nurse staffing and omissions in nursing care: A systematic review

**DOI:** 10.1111/jan.13564

**Published:** 2018-04-23

**Authors:** Peter Griffiths, Alejandra Recio‐Saucedo, Chiara Dall'Ora, Jim Briggs, Antonello Maruotti, Paul Meredith, Gary B. Smith, Jane Ball, the Missed Care Study Group

**Affiliations:** ^1^ Faculty of Health Sciences University of Southampton Southampton UK; ^2^ National Institute for Health Research Collaboration for Leadership in Applied Health Research and Care (CLAHRC) Wessex UK; ^3^ University of Portsmouth Portsmouth UK; ^4^ Portsmouth Hospitals NHS Trust Hampshire UK; ^5^ University of Bournemouth Poole UK

**Keywords:** care left undone, hospital, implicit rationing, missed care, nursing staff, quality, skill mix, systematic review, workforce

## Abstract

**Aims:**

To identify nursing care most frequently missed in acute adult inpatient wards and to determine evidence for the association of missed care with nurse staffing.

**Background:**

Research has established associations between nurse staffing levels and adverse patient outcomes including in‐hospital mortality. However, the causal nature of this relationship is uncertain and omissions of nursing care (referred as missed care, care left undone or rationed care) have been proposed as a factor which may provide a more direct indicator of nurse staffing adequacy.

**Design:**

Systematic review.

**Data Sources:**

We searched the Cochrane Library, CINAHL, Embase and Medline for quantitative studies of associations between staffing and missed care. We searched key journals, personal libraries and reference lists of articles.

**Review Methods:**

Two reviewers independently selected studies. Quality appraisal was based on the National Institute for Health and Care Excellence quality appraisal checklist for studies reporting correlations and associations. Data were abstracted on study design, missed care prevalence and measures of association. Synthesis was narrative.

**Results:**

Eighteen studies gave subjective reports of missed care. Seventy‐five per cent or more nurses reported omitting some care. Fourteen studies found low nurse staffing levels were significantly associated with higher reports of missed care. There was little evidence that adding support workers to the team reduced missed care.

**Conclusions:**

Low Registered Nurse staffing is associated with reports of missed nursing care in hospitals. Missed care is a promising indicator of nurse staffing adequacy. The extent to which the relationships observed represent actual failures, is yet to be investigated.

**Why is this research/review needed?**

- The role of nurse staffing to maintain patient safety was recognized in the safe staffing guidelines for adult hospital wards produced by the National Institute for Health and Care Excellence.
- The National Institute for Health and Care Excellence has highlighted the need for more evidence on indicators that more directly reflect the impact of nurse staffing on patient outcomes.

**What are the key findings?**

- Low Registered Nurse staffing is associated with omission of essential care.
- Missed care is a promising indicator of nurse staffing adequacy.

**How should the findings be used to influence policy/practice/research/education?**

- Given the potential consequences of missed care, its incidence/prevalence may serve as an indicator of care quality.
- Maintaining adequate staffing levels is a mechanism to avoid missed care.

## 1 INTRODUCTION

The evidence of nurse staffing levels in hospitals and its association with patient outcomes is extensive. However, several other factors may affect outcomes throughout the period a patient stays in hospital. More recently, research has begun to explore missed nursing care as a key factor leading to adverse patient outcomes. Missed care has also been identified as a plausible indicator of hospital nursing care quality (Griffiths, Ball, et al., 2014; Griffiths, Dall'Ora, et al., 2014). Worldwide predictions of a shortage of nurses by 2025, driven by retiring workforce and an ageing population, increases the need to develop a deep understanding of the impact of nurse staffing on patient safety. Furthermore, identifying the mechanisms and all possible outcomes that can be affected by unsafe staffing in hospital raises international interest.

### 1.1 Background

Low nurse staffing levels are associated with adverse outcomes in hospitals, most notably mortality (Griffiths et al., 2016; Kane, Shamliyan, Mueller, Duval, & Wilt, 2007; Shekelle, 2013). While this evidence has had significant impact and has been used to advocate for increased nurse staffing levels, including mandatory minimums, the causal link between nurse staffing levels and outcomes remains disputed (Griffiths, et al., 2016). Certainly for most patient outcomes the causal association can only be partial and indirect.

More recently, missed nursing care, defined as any aspect of care that is omitted or delayed, in part or in whole (Kalisch, Landstrom, & Hinshaw, 2009), has captured attention, with some evidence that it may be associated with adverse patient outcomes (Carthon, Lasater, Sloane, & Kutney‐Lee, 2015; Lucero, Lake, & Aiken, 2010). Enquiries into potentially avoidable deaths in hospital demonstrate how omissions by nursing staff can lead to serious adverse outcomes. For example, reports into avoidable deaths in hospital identify that a failure to measure patients’ vital signs, recognize the early signs of deterioration, communicate abnormal observations and/or provide an adequate response are frequently associated with avoidable deaths (Dagmar, Kate, & Frances, 2007).

Consequently, omission in essential care, in particular surveillance to identify and prevent deterioration, has been hypothesized as the mechanism through which mortality rates are influenced by nurse staffing levels (Clarke & Aiken, 2003). In the face of excessive workloads, nurses may be unable to complete all necessary care activities and must, in effect, engage in what is described as “implicit rationing” (Schubert, Glass, Clarke, Schaffert‐Witvliet, & De Geest, 2007). Missed nursing care has also been suggested as a potential quality measure linked to the adequacy of nursing staffing (National Institute for Clinical Excellence, 2012; VanFosson et al., 2016), acting as a leading indicator which could more sensitively indicate problems arising from low staffing before they could be detected through adverse outcomes (Ball, Murrells, Rafferty, Morrow, & Griffiths, 2014).

However, while evidence for the association between nurse staffing levels and patient outcomes is considerable and has been extensively reviewed, research on missed nursing care is more limited, in part because nursing activities can be difficult to measure and are often not routinely collected by healthcare providers (Lucero et al., 2010). However, there is now a growing number of studies exploring the link between nurse staffing and missed care. Previous reviews have considered factors related to missed care but have not systematically explored the link with staffing (Jones, Hamilton, & Murry, 2015).

## 2 THE REVIEW

### 2.1 Aims

The aim of this review was to identify nursing care tasks most frequently missed in acute hospitals’ adult inpatient wards and to determine evidence for the association of missed care with nurse staffing.

Two questions guided the review to reach the aims:

1. What are the nursing care tasks most frequently missed in acute hospitals adult inpatient wards, as reported by staff or patients or captured in administrative data?
2. What are the associations between missed care and nurse staffing levels or skill mix in acute hospitals adult inpatient wards?

### 2.2 Design

This was a systematic review of quantitative studies exploring associations between nurse staffing levels and skill mix with missed care in general medical/surgical wards in acute hospitals.

The review was conducted according to the review methods outlined in the guidance for the development of public health described by NICE (2012). This approach was selected because we anticipated that most research would be observational.

### 2.3 Search methods

Our search strategy was based on a comprehensive examination and review of literature on nurse staffing (Griffiths, Ball, et al., 2014; Griffiths, Dall'Ora, et al., 2014), supplemented by additional searches for specific terms related to missed care (missed care, unfinished care, implicit rationing, care left undone, task left undone) [see Supporting Information for full search strategy]. Searches were performed on CEA registry, CDSR, CENTRAL, CINAHL, DARE, Econlit, Embase, HTA database, Medline including In‐Process, NHS EED, HEED and databases of grey literature (including the HMIC database and those held by the National Institute for Health and Care Excellence [NICE]). Search dates were limited to studies published from 2006 when the term missed care was used in a study by Kalisch (2006). Manual journal searches, literature from personal libraries and reference lists were reviewed. Initial searches were undertaken to June 2016. Additional searching during the final drafting of the paper suggested no major new studies had been published subsequently, although we can only be confident of comprehensive coverage up to June 2016.

### 2.4 Search outcome

We included primary studies exploring the association between a measure of nurse staffing (e.g. RN Hours per patient day or nurse patient ratio) or skill mix (e.g. ratio of RN to all hands on carers) and missed care (measured as scores or frequencies of nursing tasks, procedures or aspects of care missed or substantially delayed) in acute care hospital wards. Studies undertaken exclusively in highly specialist units with atypical staffing (e.g. ICU) were excluded. Studies reporting composite error rates that might include omissions (e.g. medication errors) were excluded if the rate of omissions could not be separated from other errors. We considered quantitative randomized or non‐randomized controlled trials, prospective or retrospective observational studies, and cross‐sectional or longitudinal studies.

A single reviewer (AR) undertook initial screening of titles and abstracts for relevance. Two reviewers independently scrutinized the list of potentially relevant studies and identified studies for inclusion. Disagreements were resolved by discussion; initial discrepancy on decisions of the studies finally included was low (1 study in 18 required any discussion).

**Figure 1:**
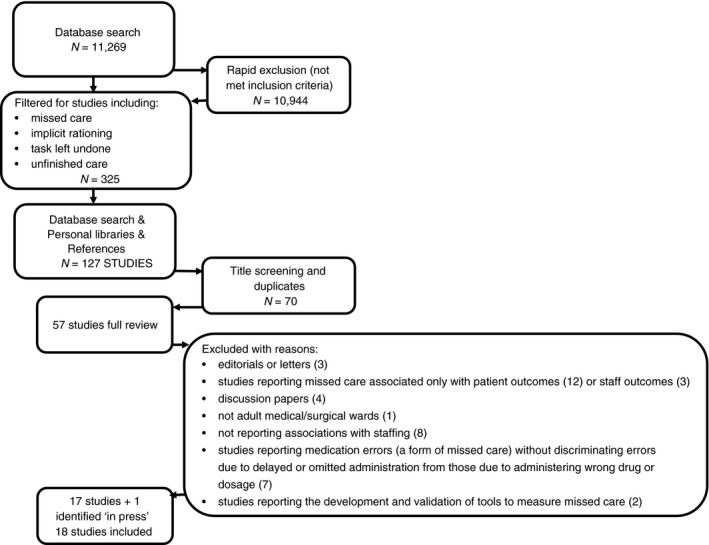
Study selection flow chart

Our searches identified 11,269 references. After deleting duplicates and rapid screening for relevance (title only), 127 studies were identified requiring further consideration. Further abstract screening resulted in 57 studies kept for full text review. Following review, 40 papers were excluded due to: type of article (e.g. discussion, review) (*N* = 7); not measuring associations with nurse staffing (*N* = 25); not adult medical/surgical wards (*N* = 1); reporting medication errors without discriminating errors due to delayed or omitted administration from those due to administering wrong drug or dosage (*N* = 7) (see Figure 1).

### 2.5 Quality appraisal

To assess the risk of bias in studies, we adapted the NICE quality appraisal checklist for quantitative studies reporting correlations and associations (National Institute for Clinical Excellence, 2012). Risk of bias was expressed in terms of internal and external validity. Internal validity included information on reliability and completeness of the measurements and ability of the study to control for potential confounding factors. External validity was assessed primarily by considering representative sampling of centres for a large region/country (e.g. random sample of hospital) and statistical power. The complete appraisal checklist is available in Supporting Information. Assessments were completed by one reviewer and corroborated independently by a second reviewer.

### 2.6 Data abstraction

Data were abstracted on study design, setting, sample characteristics (sample size of both staff and patients), staffing measures, prevalence of nursing tasks missed and measures of association/effect. Where possible we extracted full details of the association including point estimate, confidence interval and exact *p* value. Where reporting was incomplete, we included the detail provided by authors (e.g. the author's report that an association was statistically significant).

### 2.7 Synthesis

Assessment of missed care differed between instruments both in terms of aspects of missed care assessed and wording of questions. BERNCA assessed, “number of necessary nursing tasks for patients withheld or otherwise not performed in the last seven working days”, the MISSCARE survey asked, “frequency of care missed on unit by all of the staff (including yourself)” while the RN4Cast/IHOC, survey asked, “on your most recent shift, which of the following activities [13‐item list] were necessary but left undone because you lacked the time to complete them?”. Details of the items are given in Table 2. The QTDS asked patients to rate whether any of the six elements of discharge information were needed but not received.

Because of the varying approaches to measuring missed care (i.e. number of items in each instrument and reference to time period when missed care occurred), to analyse the relative frequency of missed care across studies, we rank‐ordered the frequency with which items of care were reported as missed. Where studies had reported missed care using the same instrument we then calculated the average rank of each item. Where items were rated on a scale, we considered ratings of “always” or “often” to be “missed” care. To avoid overweighting items from instruments used in only a single study we only included results from instruments if they had been used in multiple studies and/or studies with a large sample of hospitals (10+) and nurses (1,000+). This meant that results would not be unduly influenced by any studies that had been undertaken using a unique instrument using only a small sample. We coded missed care items as clinical, planning and communication or unclassified, based on the factor analysis of the RN4Cast survey (Bruyneel et al., 2015). Items were classified into one of the two factors where three of four independent raters agreed on the classification. Because different scales had different numbers of items, rankings were converted to centiles to create a comparable metric so the most scored item always scored 100 and the least scored item scored 0. Rankings were compared using Kruskall–Wallis (non‐parametric) Analysis of variance (Minitab v 17.3).

For associations between staffing/skill mix, we adopted a narrative approach to synthesis because of the diversity of settings, measures of staffing and missed care, and approaches to analysis reported, which rendered statistical meta‐analysis meaningless. Our narrative synthesis was guided by a “vote counting” approach based on significant results and weighted towards larger and higher quality studies.

## 3 RESULTS

### 3.1 Included studies

In total, eighteen studies met the inclusion criteria for this review. The external validity of seven studies was assessed as strong (Ausserhofer et al., 2014; Ball et al., 2014; Ball et al., 2016; Bruyneel et al., 2015; Griffiths, Dall'Ora, et al., 2014; Schubert et al., 2013; Zander, Dobler, Baumler, & Busse, 2014), largely because of large nationally representative samples of hospitals and nurses, all part of the multinational RN4Cast study. All studies had at least moderate risk of bias (internal validity). Table 1 presents a summary of the included studies.

**Table 1:** Included studies

Study	Country	Hospitals	Unit type(s)	Participants	*n*	Missed care measure	Validity^b^		Effect of nurse staffing^c^(summary)	Main results
							Internal	External		
Al‐Kandari and Thomas (2009)	Kuwait	5	Med/surg	RN	780	IHOC survey	−	+	+	Significant positive correlation between the number of RNs in unit and adequate documentation of nursing care (*p* < .005)
Ausserhofer, Zander, Busse, Schubert, De Geest, Rafferty, and Consortium (2014)^a^	Europe	488	Med/surg	RN	33659	RN4CAST	+	++	+	Fewer nursing care tasks left undone in hospitals with lower p:n ratios and more favourable work environment (β = 0.09; *p*<.0001).
Ball, et al. (2014)^a^	England	46	Med/surg	RN	2,917	RN4CAST	+	++	+	Fewer patients per nurse fewer elements of care missed (*p* < .001; OR = 0.34, 95% CI 0.22–0.53).
Ball, et al. (2016)^a^	Sweden	79	Med/surg	RN	10,174	RN4CAST	+	++	+	Fewer than four patients per RN reduced the odds of missed care by 85% (OR 0·148, *p* < ·001). Compared with shifts of 10+ patients per RN, odds of missed care more than halved on shifts of six or fewer patients per RN (*p* < ·001, OR 0·466).
Bruyneel, et al. (2015)^a^	Europe	217	Med/surg	RN	10733	RN4CAST	+	++	+	Fewer clinical care tasks left undone (hypothesized hospital‐level mediator) was significantly associated with lower p:n ratio 0.039 CI (credibility intervals) [0.002, 0.072].
Cho, Kim, Yeon, You, and Lee (2015)	Korea	1	General hospital (NS)	RN	232	MISSCARE	−	−	+	High staffed units (patient per nurse ratio 7:1) were associated with less missed nursing care than low staffed units (patient per nurse ratio 17:1) (β 0.136; *p* = .02)
Cho, Lee, Kim, Kim, Lee, Park, and Sung (2016)	Korea	51	General hospital (all)	RN	3,037	RN4CAST	+	+	+	One additional patient per RN was associated with a 3% increase in the odds of care left undone (OR = 1.03 *p* < .001)
Dabney & Kalisch (2015)	USA	2	General hospital (NS)	Patient	729	MISSCARE patient	−	−	+	Patients’ reports of not receiving timely care weakly significantly correlated with RN hours per patient day (*r* = −.14, *p* = .002)
Friese, Kalisch, and Lee (2013)	USA	9	Oncology	RN & HCSW	2,318	MISSCARE	−	−	+	Increase in the number of patients cared for by RN and HCSW was associated with a 2.1% increase in the total missed nursing care score (*p* < .05)
Griffiths, Dall'Ora, et al., 2014, a	Europe	488	Med/surg	RN	31,627	RN4CAST	+	++	+	Odds of nurses leaving care undone increased by 26% when nurses were caring for >11.5 patients, compared with nurses caring for ≤6 patients (OR = 1.26; 95% CI = 1.23–1.29)
Kalisch, Tschannen, Lee, and Friese, 2011	USA	10	General hospital (all)	RN & HCSW	4,086	MISSCARE	+	+	+	Staff that cared for more patients reported more missed care (β = 0.015 *p* < .001)
Kalisch, Tschannen, and Lee, 2011	USA	10	General hospital (all)	RN & HCSW	4,288	MISSCARE	+	+	+	Higher nursing hours per patient day were associated with lower level of missed care (β = −0.45 *p* = .002)
Kalisch, Doumit, Lee, and Zein (2013)^c^	USA/Lebanon	2	Med/surg/ICU	RN	633 114	MISSCARE	−	−	(+)	Number of patients cared for was not a significant predictor of missed care
Orique, Patty, Woods (2015)	USA	1	General hospital (all)	RN & HCSW	169	MISSCARE	−	−	+	No significant relationship between unit‐level nurse workload and missed nursing care but significant positive relationship between missed care and number of patients under care (*r*_ _= .246, *p* = .001)
Palese, Ambrosi, Prosperi, Guarnier, Barelli, Zambiasi, and Saiani (2015)	Italy	12	Med	RN & HCSW	205 109	MISSCARE	+	+	+	Lower p:n was associated with less missed care (OR = 0.91 *p* < .05)
Schubert, Ausserhofer, Desmedt, Schwendimann, Lesaffre, Li, and De Geest (2013)^a^	Swiss	35	Med/surg	RN	1,633	BERNCA revised	+	++	+ (+)	Shifts with ≤6 patients per RN were associated with a 53% reduction in the odds of care left undone, compared with shifts on which there were ≥10 patients per RN (OR = 0.47, *p* < .001).
Weiss, Yakusheva, and Bobay (2011)	USA	4	Med/surg	Patient	1,892	QTDS (delivered)	+	+	(−)	No significant association between non‐overtime RNHPPD and patient reported delivery of necessary discharge information (β −0.05 *p* = .74).
Zander, Dobler, Baumler, and Busse (2014)^a^	German	49	Med/surg	RN	1,511	RN4CAST	+	++	+ (+/−)−	Missing patient surveillance, skincare and medication given on time were increased by 3% for each additional patient per nurse (OR = 1.03 *p* < .01). Some aspects of care showed smaller but significant associations in the opposite direction; no overall relationship.

a Studies that are based on the data collected as part of the RN4Cast study in 12 European countries Belgium, England, Finland, Germany, Greece, Ireland, The Netherlands, Norway, Poland, Spain, Sweden and Switzerland. Where “Europe” is indicated the study analysis was conducted by pooling data across some or all of these countries.

b − High risk of bias—few criteria fulfilled conclusions likely to be affected. + moderate risk of bias—several criteria fulfilled conclusions unlikely to be affected. ++ low risk of bias = most criteria fulfilled conclusions very unlikely to be affected.

c + Statistically significant association showing a benefit (reduced missed care from higher staffing levels). − statistically significant effect showing harm (increased missed care with higher staffing levels). (+) non‐significant association showing direction of effect or no effect.

NS, specialities not specified. p:n patients per nursing staff (RN only or RN + HCSW).

All the studies were cross‐sectional. Sample sizes ranged from 232‐31,627 nurses, most including only Registered Nurses (RNs) although some included healthcare support workers (HCSW) such as nursing assistants or licensed vocational nurses in the nursing workforce. Staffing levels were reported as patient:nurse ratio (12 studies), nursing hours per patient day (NHPPD or RNHPPD) (three studies) or number of patients cared for in last shift (three studies). Four studies explored aspects of skill mix (Ball et al., 2014; Ball et al., 2016; Dabney & Kalisch, 2015; Palese et al., 2015). Seven studies used data derived from the RN4Cast study, with four single country analyses from England (Ball, et al., 2014), Germany (Zander et al., 2014), Sweden (Ball, et al., 2016) and Switzerland (Schubert et al., 2013) plus three multi‐country analyses (Ausserhofer et al., 2014; Bruyneel, et al., 2015; Griffiths, Dall'Ora, et al., 2014). Although these papers report on separate aspects, there is considerable overlap and single country data are nested in the multi‐country analyses and so these seven studies are not fully independent.

In all studies missed care was assessed by a survey completed by nurses or, for two studies, patients (Dabney & Kalisch, 2015; Weiss, Yakusheva, & Bobay, 2011). Eight studies (Cho, Kim, Yeon, You, & Lee, 2015; Dabney & Kalisch, 2015; Friese, Kalisch, & Lee, 2013; Kalisch, Tschannen, & Lee, 2011; Kalisch, Tschannen, Lee, & Friese, 2011; Kalisch et al., 2013; Orique et al., 2015; Palese et al., 2015) reported using a version of the MISSCARE survey (Kalisch & Williams, 2009); seven studies (Al‐Kandari & Thomas, 2009; Ausserhofer, et al., 2014; Ball, et al., 2016; Ball, et al., 2014; Bruyneel, et al., 2015; Griffiths, Dall'Ora, et al., 2014; Zander et al., 2014) used a survey developed for the Registered Nurse Forecasting (RN4Cast) study (Sermeus et al., 2011). One used the Basel Extent of Rationing Nursing Care Assessment (BERNCA) revised (Schubert et al., 2007), a version of the International Hospital Outcomes Survey (IHOS) (Al‐Kandari & Thomas, 2009) and one the Quality of Discharge Teaching Scale (QTDS) content delivered sub‐scale (Weiss et al., 2011). All these measures cover a broad range of clinical and psychosocial nursing care with the exception of the QTDS which focuses on content of discharge teaching. All these studies relied on recall and subjective judgement of quantities and frequency of missed care. We found no studies where objectively recorded aspects of missed care, for example, records of completed assessments of vital signs, were used.

### 3.2 Prevalence of missed care

An overall estimate of the frequency with which care was missed could be made from seven studies (Al‐Kandari & Thomas, 2009; Ball, et al., 2016; Ball, et al., 2014; Cho et al., 2016; Griffiths, Ball, et al., 2014; Griffiths, Dall'Ora, et al., 2014; Schubert, et al., 2013; Zander, et al., 2014). In European studies using the RN4Cast survey estimates of the frequency with which some care was left undone on the last shift ranged from 75% in England (Ball, et al., 2014) to 93% in Germany (Zander, et al., 2014) with an overall estimate of 88% across 12 European countries (Griffiths, Ball, et al., 2014; Griffiths, Dall'Ora, et al., 2014). Using the same instrument, a rate of 81% was reported from Korea (Cho et al., 2016). Fifty‐five per cent of nurses in Kuwait identified that they were unable to complete all required procedures on their last shift (Al‐Kandari & Thomas, 2009). Schubert et al. reported that 98% of Swiss nurses had omitted at least one item from the BERNCA survey in the last 7 days (Schubert, et al., 2013).

Several studies explored specific aspects of missed care, with each instrument addressing different aspects of care against differing denominators and with different levels of specificity, making direct comparison difficult. Table 2 presents a summary of the care missed and the relative frequency (ranked average frequency) using the three most widely used instruments. Care classified as “planning and communication” (median rank 20th centile) was reported as missed more often than clinical care (median rank 65th centile *df* = 2; *p* = .001).

**Table 2:** Relative frequency of missed care reported using three common instruments (based on average rank in studies)

Instrument											
BERNCA Revised (1 study)	Type^a^	Rank	Centile^b^	MISSCARE (6 studies)	Type	Rank	Centile	RN4CAST (5 studies)	Type	Rank	Centile
Emotional & psychological support	p	1	0%	Ambulation three times per day or as documented	u	1	0%	Comfort/talk with patients	p	1	0%
Assessment of newly admitted patient	u	2	3%	Mouth care	c	2	4%	Educating patients and family	p	2	8%
Setup care plans	p	3	6%	Attend interdisciplinary care conferences whenever held	P	3	9%	Adequately document nursing care	u	3	17%
Necessary conversation	p	4	10%	Turning patient every 2hrs	c	4	13%	Oral hygiene^a^	c	4	25%
Mobilization	u	5	13%	Patient teaching	P	5	17%	Develop or update nursing care plans/care pathways	p	5	33%
Documentation & evaluation of the care	u	6	16%	Medications administered within 30 min before or after scheduled time	c	6	22%	Frequent changing of patient position^a^	c	5	33%
Activating or rehabilitating care	u	7	19%	Full documentation of all necessary data	u	6	22%	Prepare patients and families for discharge	p	7	50%
Information about therapies	p	8	23%	Feeding patient when the food is still warm	u	8	30%	Planning care	p	8	58%
Monitoring of confused patients and use of sedatives	c	8	23%	Assist with toileting needs within 5 min of request	u	8	30%	Adequate patient surveillance^a^	c	9	67%
Studying care plans	u	10	29%	Emotional support to patient and/or family	P	10	39%	Administer medications on time^a^	c	10	75%
Monitoring patients as the nurse felt necessary	c	11	32%	Assess effectiveness of medications	c	11	43%	Pain management^a^	c	11	83%
Keep patient waiting who rung	u	12	35%	Patient bathing/skincare	c	12	48%	Skin care^a^	c	12	92%
Monitoring of confused patients & use of restraints	c	13	39%	Response to call light is provided within 5 min	u	13	52%	Treatments and procedures^a^	c	13	100%
Oral hygiene	c	14	42%	Monitoring intake/output	c	14	57%				
Delay in measure because of a physician delay	u	15	45%	Setting up meals for patients who feed themselves	u	15	61%				
Dental hygiene	c	16	48%	PRN medication requests acted on within 5 min	c	16	65%				
Continence training (diapers)	u	17	52%	IV site care and assessment according to hospital policy	c	17	70%				
Administration of medication, infusions	c	18	55%	Skin/wound care	c	18	74%				
Monitoring patients as prescribed by physician	c	19	58%	Hand washing	c	19	78%				
Skin care	c	20	61%	Ensuring patient discharge planning	P	20	83%				
Change in the position	c	21	65%	Focused reassessment according to patient condition	c	21	87%				
Preparation for discharge	p	22	68%	Patient assessment performed each shift	c	22	91%				
Sponge bath	u	23	71%	Vital signs assessed as ordered	c	23	96%				
Assist food intake	u	24	74%	Bedside glucose monitoring as ordered	c	24	100%				
Education and training	u	25	77%								
Adequate hand hygiene	c	26	81%								
Partial sponge bath	u	27	84%								
Change in wound dressings	c	28	87%								
Preparation for test and therapies	c	29	90%								
Necessary disinfection measures	c	30	94%								
Change in the bed linen	u	31	97%								
Continence training (insert catheter)	c	32	100%								

a Classification of missed care item: p, planning and communication, c, clinical care, u, unclassified (no consensus).

b Percentile rank—the original items were ranked on a scale 1 to n, where n is the number of items on the scale. To ensure comparability in item rankings across scales with different item numbers these are converted to a percentile scale where the highest ranked item (1) is 0% and the highest ranked (n) is 100%.

Using the BERNCA instrument (one study), emotional and psychological support was reported as most often missed (rank 1/32 items). Forty‐one per cent of nurses in Switzerland identified this as a task that was sometimes or often “withheld” due to inadequate staffing in the past 7 days (Schubert, et al., 2013). Comforting/talking to patients was the item most often reported as left undone on the last shift in studies using the RN4CAST survey (rank 1/13 across five studies). Frequency of reports ranged between 46% in England (Ball, et al., 2016) and 82% in Germany (Zander, et al., 2014). Conversation (4/32 BERNCA) and education/counselling patients and family (2/13 RN4cast) were also among the most frequently reported aspects of care missed in these studies. The most frequently missed aspect of care reported on the MISSCARE survey was patient ambulation (ranked 1/24). This was reported as always, frequently or occasionally missed by between 76% of nurses in the USA (Kalisch, Tschannen, Lee, Friese, 2011) and 91% of nurses in Italy (Palese, et al., 2015). Other commonly missed elements of care included assessment of newly admitted patients (2/32) and set up of care plans (3/34) [BERNCA], mouth care (2/24) and attending interdisciplinary care conferences (3/24) [MISSCARE] and documenting nursing care (3/13) [RN4CAST]. In general, where equivalent or similar items appeared on other instruments these items also ranked in the top half of care most frequently missed.

While clinical care was less often reported as missed, the rates of omission were still substantial. Monitoring of vital signs was one of the least likely aspects to be reported as missed in studies using MISSCARE (rank average 23/24), however, omissions of care in this area were still relatively frequent, ranging between 16% (Palese, et al., 2015) and 35% (Palese, et al., 2015). Studies using the RN4CAST measure found up to 37% of nurses reporting some care missed on the last shift (Zander, et al., 2014) while “patient monitoring as prescribed by the physician” was reported as often/sometimes missed by 17% of nurses using BERNCA (Schubert, et al., 2013).

### 3.3 Associations with staffing levels

Of the 18 studies, 14 found that lower nurse staffing levels were significantly associated with higher levels of missed nursing care. Two studies (Kalisch et al., 2013; Schubert, et al., 2013) showed no significant effects (Table 1 for a summary of significant effects and direction of association) and two showed mixed effects. Across all studies only two showed any association in the opposite direction—in one case non‐significant and in the other cases, results showed associations in different directions for different aspects of missed care.

In the RN4CAST study of 31,627 RNs from 488 hospitals in 12 European countries, the odds of nurses leaving care undone were increased by 26% when nurses were caring for >11.5 patients, compared with nurses caring for ≤6 patients (OR = 1.26; 95% CI = 1.23–1.29) (Griffiths, Ball, et al., 2014; Griffiths, Dall'Ora, et al., 2014). Two further multi‐country analyses from the same study using the number of care items missed as the outcome confirmed this significant association (Ausserhofer, et al., 2014; Bruyneel, et al., 2015). Of the four single country analyses based on these data, statistically significant associations between lower staffing and higher levels of missed care were found in England (Ball, et al., 2014) and Sweden (Ball, et al., 2016), but results from Germany (Zander, et al., 2014) and Switzerland (Schubert, et al., 2013) were more equivocal. In England, odds of missing care were 66% lower when RNs were caring for 6.1 patients or fewer, compared with nurses caring for 11.7 or more patients (OR = 0.34, 95% CI 0.22–0.53). In Sweden, shifts with ≤6 patients per RN were associated with a 53% reduction in the odds of care left undone, compared with shifts on which there were ≥10 patients per RN (OR = 0.47, *p* < .001). In Germany, reports of missing patient surveillance, skincare and medication given on time were each increased by 3% for each additional patient per nurse (OR = 1.03 *p* < .01) although for some aspects of care there were smaller but significant associations in the opposite direction, suggesting no overall relationship. However, in Switzerland the patient:nurse ratio was significantly associated with missed care only when using an unadjusted model. When analysis was adjusted for possible confounders, the association was no longer significant although there was a significant association with nurse‐perceived staffing adequacy.

Similar results were found in other countries. In South Korea, a cross‐sectional survey with 3,037 RNs, found that every one additional patient per RN was associated with a 3% increase in the odds of care left undone using the RN4CAST measure (OR = 1.03; *p* < .001) (Cho, et al., 2016), high staffed units (1:7, nurse:patient ratio) were associated with less missed nursing care than low staffed units (1:17, nurse:patient ratio) (β = 0.136; *p* = .02) (Cho et al., 2015) and a study in 12 Italian hospital units, found that a lower number of patients cared for by each RN was associated with less missed care (OR = 0.91; *p* < .05) (Palese, et al., 2015). Weak negative correlations between completion of most nursing tasks and staffing levels were observed in Kuwait, with the strongest correlation (*r* = −.12) between the total workload and completion of teaching patient/family (*p* < .005) (Al‐Kandari & Thomas, 2009). All these studies rated as moderate or high risk of bias.

In the USA, a study of 4,086 nursing staff in 10 hospitals found that staff that cared for more patients reported more missed care (β = 0.015; *p *< .001). (Kalisch, Tschannen, Lee, and Friese, 2011). A second found that higher nursing hours per patient day were associated with lower level of missed care (β = −0.45; *p *= .002). (Kalisch, Tschannen, Lee, 2011). These studies were rated as having moderate risk of bias, but we could not ascertain if these were fully independent studies. Smaller and lower quality studies provided a similar but more mixed picture. A study in oncology units in nine hospitals found a 1‐patient increase in the number of patients cared for by RNs and HCSW was associated with a 2.1% increase in the total missed nursing care score (*p* < .05) (Friese et al., 2013), and a single hospital study found a weak positive correlation between the number of patients per nurse and missed care (*r* = .246, *p* < .001). (Palese, et al., 2015). However, a study in two hospitals in the USA and Lebanon (Beirut) found the number of patients cared for was not a significant predictor of missed care (Kalisch, Doumit, Lee, and Zein, 2013).

Mixed results were found in studies using patient reports, although these studies had a high or moderate risk of bias. A study of 729 patients in two US hospitals found that patients’ reports of not receiving timely care was weakly correlated with RN hours per patient day (*r* = −.14, *p* = .002) (Dabney & Kalisch, 2015) but found no correlation with overall patient reports of missed care. A study focussing on discharge planning in four US hospitals found no significant association between non‐overtime RNHPPD and patient‐reported delivery of necessary discharge information (β = −0.05, *p* = .74) (Weiss et al., 2011).

### 3.4 Skill mix and missed care

Four studies (Ball, et al., 2014; Ball, et al., 2016; Dabney & Kalisch, 2015; Palese, et al., 2015) explored associations between skill mix and missed care either directly or indirectly. The results suggest that adding support workers to the workforce does not generally reduce the level of missed nursing care and may even increase it where skill mix is diluted. Patient‐reported timeliness of care was significantly correlated with increased RN skill mix in one study (*r* = .13, *p* < .001) (Dabney & Kalisch, 2015). Another study found that more daily care provided by support workers was associated with increased nurse‐reported frequency of missed care (OR = 1.04; 95% CI = 1.01–1.07) (Palese, et al., 2015), while two studies found that higher numbers of support workers were not associated with reductions in the rate of care left undone except with very high levels of support worker staffing in one study (<4 patients per support worker OR = 0.71, *p* = .021) (Ball, et al., 2014; Ball, et al., 2016).

## 4 DISCUSSION

Our review found that the relationship between “missed care” and nurse staffing levels in hospitals has been widely studied in many countries. However, all of the research that we identified used subjective measures of missed care, with most relying on retrospective reports by nurses. Overall reported levels of missed care were high. Aspects of planning and communication were more likely to be reported as missed than clinical care, although reports of missing essential clinical procedures and patient monitoring/observation were still common. Almost all studies found that low nurse staffing levels were related to higher reports of missed care. The findings from most studies related to RN staffing levels. Evidence related to the skill mix of the nursing team pointed to either no benefit or a negative effect from higher levels of support workers although a single study found that when HCSW staffing levels were very high there was a reduced level of missed care.

Interest in missed care nursing has often been based on its role as a potential mechanism explaining the association between patient safety outcomes and nurse staffing levels. The hypothesis that low staffing contributes to high mortality through missed opportunities to identify and prevent deterioration is an important part of the argument that these associations between staffing and outcomes are causal (Clarke & Aiken, 2003; Griffiths, et al., 2016). The evidence in this review does lend some support to this claim although the current subjective measures, based on intermittent survey, do not readily lend themselves to routine quality monitoring despite recent interest in using missed care as a leading quality indicator.

The care most frequently reported as missed, such as talking to and comforting patients, is of intrinsic importance to patients but it is unlikely to directly explain how staffing levels influence adverse outcomes such as mortality. The relative neglect of these aspects of care may indeed reflect prioritisation of clinical care in the face of staff shortages for sound clinical reasons although less positive motivations, including deference to medicine, have also been suggested (Papastavrou et al., 2014). Nonetheless, the levels of omission of clinical care reported are still substantial. Broad measures of missed care make no distinction between the relative importance of the care that is missed. Not all missed care is of equal significance and impact on patient outcomes will vary (Recio‐Saucedo et al., 2017). While there is some evidence that missed care mediates the relationship between staffing levels and measures of patient satisfaction (Bruyneel, et al., 2015) and falls (Kalisch et al., 2012), direct evidence of omitted clinical care mediating the relationship between staffing and mortality is absent (Jones et al., 2015).

Furthermore, while the studies reviewed here generally support the association between staffing levels and missed care, none tested an association between staffing levels and any objective measure of care. Although nurse reports of missed care are associated with adverse patient outcomes (Recio‐Saucedo, et al. 2017), it remains unclear to what extent these reports correlate with actual omissions of care. While studies that failed to show significant associations were typically smaller and had a higher risk of bias, those which used patient reports did not clearly confirm the results of studies which used nurse reports. The differences in measures and approaches to analyses used make comparison across studies difficult. Extrapolating from the results of one of the studies with the most “dramatic” appearing effect size, (Ball, et al., 2016) the reported 66% reduction in the odds of reporting missed care in the best staffed wards compared with the worst equates to a reduction from 89‐75%. It seems that although staffing levels may have an association with the rate of reported missed care, most missed care cannot be attributed to low staffing.

### 4.1 Limitations

While our review was based on an extensive search strategy it is possible that we could have missed studies that focussed on omissions of very specific aspects of care. However, this literature would have to be extensive to change our broad conclusions. There may have been publication bias in relation to staffing outcome associations. However, because the associations were supported by very large studies, again unpublished evidence would have to be extensive to substantially alter our results. While we have relied on the conclusions from some studies where the reporting of results was incomplete, our conclusions would only be changed if the reporting of results by authors was systematically wrong in several studies. Our conclusions are based exclusively on cross‐sectional studies. While the link to nursing work is direct, making a causal interpretation plausible, associations in these studies cannot establish causation. Our quality appraisal instrument was designed to assess relative quality of cross‐sectional studies following the methodology of NICE (National Institute for Clinical Excellence, 2012) and therefore assigning a low risk of bias to a study does not assure a causal interpretation.

## 5 CONCLUSION

While reported missed care is associated with nurse staffing levels and such reports may indeed be indicators of inadequate nurse staffing, there is no research demonstrating associations with objective measures of care. The extent to which the relationships observed in these studies represent actual omissions of care and the consequences of such failures, remains largely uninvestigated. Given the potential consequences of missed care, its incidence/prevalence may serve as an indicator of care quality and maintaining adequate staffing levels is a mechanism to avoid missed care. While the association between staffing and missed care is substantial it is unlikely that most care omissions are directly linked to staffing levels only. Reports of missed care cannot in themselves be used to track nurse staffing adequacy, although changes in the rate or frequency or reports could indicate nurse staffing problems. Future research should focus on objective measures of missed care on patient outcomes.

## CONFLICT OF INTEREST

No conflict of interest has been declared by the authors.

## AUTHOR CONTRIBUTIONS

All authors have agreed on the final version and meet at least one of the following criteria [recommended by the ICMJE (http://www.icmje.org/recommendations/)]:

- substantial contributions to conception and design, acquisition of data, or analysis and interpretation of data;
- drafting the article or revising it critically for important intellectual content.
